# A Study on Presentation Attack Detection in Thermal Infrared

**DOI:** 10.3390/s20143988

**Published:** 2020-07-17

**Authors:** Marcin Kowalski

**Affiliations:** Institute of Optoelectronics, Military University of Technology, 2 Gen. S. Kaliskiego St., 00-908 Warsaw, Poland; marcin.kowalski@wat.edu.pl

**Keywords:** thermal face spoofing detection, presentation attack detection, thermal infrared, facial counter spoofing

## Abstract

Face recognition systems face real challenges from various presentation attacks. New, more sophisticated methods of presentation attacks are becoming more difficult to detect using traditional face recognition systems. Thermal infrared imaging offers specific physical properties that may boost presentation attack detection capabilities. The aim of this paper is to present outcomes of investigations on the detection of various face presentation attacks in thermal infrared in various conditions including thermal heating of masks and various states of subjects. A thorough analysis of presentation attacks using printed and displayed facial photographs, 3D-printed, custom flexible 3D-latex and silicone masks is provided. The paper presents the intensity analysis of thermal energy distribution for specific facial landmarks during long-lasting experiments. Thermalization impact, as well as varying the subject’s state due to physical effort on presentation attack detection are investigated. A new thermal face spoofing dataset is introduced. Finally, a two-step deep learning-based method for the detection of presentation attacks is presented. Validation results of a set of deep learning methods across various presentation attack instruments are presented.

## 1. Introduction

The presentation attack detection technology aims to determine whether the current subject is authentic. A variety of presentation attacks are well known [1] including direct presentation attack at a sensor level. Attacks on facial sensors are presumably the easiest attacks to be performed since they could be done using relatively cheap and accessible instruments including printed or displayed photographs.

This paper presents results of a study on presentation attack detection (PAD) in thermal infrared using simple two-dimensional attacks, as well as novel 3D facial masks. The study aims to analyze the impact of varying conditions on PAD performance. For the purpose of this study long lasting experiments have been performed to assess the impact of thermalization. Images of genuine subjects after the physical effort have been used to introduce a larger variation of the dataset. In this study, a wide range of presentation attack instruments has been used including printed and displayed attacks, 2D paper-printed masks, 3D-printed masks, custom flexible latex full-face masks and custom silicone masks. Thermal images of subjects’ faces are collected in a long lasting scenario to evaluate thermalization impact of presentation attack detection, as well as individual heat patterns emitted by a human face are analyzed. In order to assess the impact of imagers’ parameters on PAD performance, studies are carried out using high-end and low-cost thermal imagers. Based on the collected database a numerical analysis has been conducted. Distribution of thermal radiation emitted by the face is analyzed, in particular emission differences between specific landmarks of the face and neck. Finally, a deep learning method for a non-intrusive PAD based on distribution of thermal energy emissions has been proposed. A two-step method consists of a head detection algorithm and a neural network classifier. Validation results of 16 different algorithms with two external datasets are presented.

The contributions are summarized as follows:

- Thermal face spoofing dataset (TFSD) of thermal images presenting attacks using printed photograph, displayed photograph, 3D-printed facial mask and full-face latex masks;
- Presentation attack detection method based on a combination of the head-detection algorithm and deep neural network-based classifiers;
- Comparison of the results with existing studies in the PAD field;
- Spectral characterization of facial masks in the thermal infrared domain;
- Study of the thermalization impact, as well as varying the subject’s state due to physical effort, on presentation attack detection are investigated;
- Results of cross-material validation for a wide range of presentation attack instruments (PAIs) and 16 different deep learning models.

In Section 2 a brief review of related works is provided. The theoretical analysis of thermal phenomena, as well as the measurement protocol are described in Section 3, and the image analysis is presented in Section 4. The PAD method is described in Section 5. Finally, the validation methodology with results and a summary of this study are provided in Section 6 and Section 7, respectively.

## 2. Related Works

PAD technology can be classified as an intrusive or a non-intrusive detection technology. In the intrusive presentation attack detection method, a user needs to cooperatively complete some specific live actions such as blinking, head turning or mouth opening. While performing facial recognition based on given instructions, the presentation attack detection module can determine whether an operator accurately completes the live operation and whether that operator is an authentic user.

In the non-intrusive presentation attack detection technology the user does not need to cooperatively complete any live action, so that the user’s experience is better, but the technical complexity is much higher. Moreover, during the non-intrusive presentation attack detection, the sensor is aimed to provide sufficient information and an image analysis is performed to detect attempts to change the face appearance. A variety of methods based on a visible range image analysis has been proposed, however their performance still can be improved, especially in terms of detecting more sophisticated attacks.

Most of the works on face presentation attack detection rely on visible or near infrared spectrum, including several datasets publicly available: NUAA [2], ZJU Eyeblink [3], Idiap Print-attack [4], Idiap Replay-attack [5], CASIA FASD [6], MSU-MFSD [7], MSU RAFS [8], 3DMA [9] or UVAD [10]. Other spectrums, including thermal infrared, have not been explored in details. There are not enough presentation attack detection datasets composed of thermal images. However, new datasets appeared presenting spoofed samples in other spectrums, including thermal infrared.

Researchers use various spoofing items or presentation attack instruments (PAIs) to perform an attack on the face recognition system. The most extensively explored PAIs include a printed photo or replaying a video of a subject. However, more sophisticated PAIs are appearing, including 3D-printed masks, facial silicone masks or full-face latex masks. Multispectral latex mask-based video face presentation attack (MLFP) database [11] contains the videos in visible, near-infrared, thermal spectrum, custom silicone mask attack dataset (CSMAD) contains thermal images of faces wearing custom silicone masks [12] and a wide multi-channel presentation attack [13].

A range of processing methods has been proposed with a vast majority operating in the visible spectrum. Many algorithms use specifically tailored features, which may be heavily pertinent to the kind of attack being detected by focusing on the texture analysis [14], motion analysis [15], life sign indicators [16] and 3D properties [17], also with a variety of machine learning based methods. Detection of 3D facial masks has been addressed in several works. Most of the works reported in this field present advances based on the exploration of visible or near infrared spectrum. Current efforts are focused on new methods that will work robustly on unknown attacks. Generalization aspect seems to be one of the biggest challenges for current PAD systems.

Erdogmus et al. [18] released the first 3D mask attack dataset (3DMAD). The dataset has been composed of images in 2D visible and 3D images presenting subjects wearing 3D face masks from Thatsmyface. In this study, the block-based local binary patterns (LBP) features with linear discriminant analysis (LDA) give best results with both RGB and 3D depth images, for which Half Total Error Rate (HTER) values are found to be of 0.95% and 1.27%, respectively. Liu et al. [19] presented a remote photoplethysmography (rPPG)-based method to detect 3D masks. This method achieved an area under the curve (AUC) of 91.7% with equal error rate (EER) of 16.2%. In another work, Li et al. [20] proposed a 3D mask detection method based on pulse detection. Liu et al. [19] and Li et al. [20] mainly analyze different heartbeat signals between real faces and 3D, and are sensitive to camera settings and variable light conditions. Bhattacharjee et al. [12] explored vulnerability of the convolutional neural network (CNN)-based face-recognition systems to presentation attacks performed using custom-made silicone masks. A comparative study [12] revealed that convolutional neural network (CNN)-based face-recognition systems are vulnerable to various 3D masks attacks. The impostor’s attack presentation matches rate for having been calculated for FaceNet, LightCNN and VGG-Face with the values of 22.6%, 42.1% and 57.9%, respectively. Shao et al. [21] presented a 3D mask detection method based on motion optical flow features extracted from a pretrained VGG-net. Manjani et al. [22] presented a silicone mask detection method based on a deep dictionary learning with a greedy learning algorithm for the optimization problem. The proposed method obtained HTER’s ranging from 14.1% to 44.8%.

Presentation attack detection in thermal infrared has not yet achieved high popularity among researchers. Thermal spectrum has been explored for face recognition techniques [1,17] however, thermal presentation attack (PA) detection is less popular. The main reason of PAD’s less popularity in thermal infrared domain is the cost of sensors and a still relatively small number of images’ scientific datasets. Visible light sensors are more accessible with prices starting from tens of dollars comparing to the thermal infrared sensors that cost at least several thousand dollars. During the presented study, a state-of-the-art thermal imager from FLIR Inc. (Wilsonville, OR, USA) as well as low-cost imager from Seek were used. Utilization of these two imagers should reveal the impact of imagers’ parameters on PAD performance. The number of PA datasets in the visible domain is considerably higher than the thermal image datasets. Sun et al. [23] proposed a liveness detection approach based on thermal infrared and visible spectra. The detection method uses a canonical correlation analysis between the visible and thermal face. The results show that the PCCW-CCA method obtains a live detection rate of 85.1% and 90.8% on data, including and excluding glasses with a false acceptance rate of 0.1%. Other methods include a thermal face-convolutional neural network (Thermal Face-CNN) with external knowledge [24]. The external knowledge used in the proposed method relies on temperature that a human face can have. The face liveness detection relies on absolute temperatures registered by a thermal camera fused with visible range images. However, relying on temperature values is valid to a limited extent, since temperature may vary depending on the environment conditions and emissivity. The proposed method obtained the best accuracy of 0.7918 with a recall of 0.7434 and a precision of 0.8298. George et al. [13] proposed a multi-channel convolutional neural network (MC-CNN) tested with grayscale, depth, infrared and thermal infrared images. The proposed method was reported to obtain an average classification error rate (ACER) of 2.59% and 0.84% for thermal imaging and a combination of grayscale, depth, infrared and thermal, respectively. This method obtains high results when using different channels and should not be considered in a single-channel configuration.

This work is the first one presenting such a wide range of PAIs including simple 2D presentation attacks and 3D masks with two different thermal imagers of a different performance. This paper is the only work presenting an impact of the variable subject’s state and heat transfer between the subject’s face and the mask on PAD performance. While most of the methods focus on developing new algorithmic approaches for PAD, this study proposes a complex approach for detecting 2D and 3D attacks that could be linked with any algorithm. During this study sixteen state-of-the-art deep learning algorithms were investigated and compared.

## 3. Measurement Process

### 3.1. Spectral Properties

A thermal camera is considered as a face presentation attack detector due to its ability to assign a quantified amount of thermal energy to the captured subject. Thermal image of the human face presents its unique heat-signature, which can be used as a pattern for recognition [25]. Presentation attack detection might be achieved with a thermal infrared camera based on an analysis of the relative temperature distribution on the surface of the face and neighboring areas. Thermal imaging relies on passive emissions and it does not need illumination. Radiation registered is proportional to the relative distribution of apparent temperature of objects placed in the field of the camera view. Objects’ projection depends on parameters of camera and lens, as well as on temperature difference between objects and their emissivity. Image intensities also strongly depend on environmental conditions of the measurement.

Ability to capture and quantify thermal energy depends on camera’s parameters. The noise equivalent temperature difference (NETD) directly defines the camera ability to detect small differences of temperatures, thus detecting the subject’s shape. Infrared cameras equipped with uncooled microbolometer focal plane arrays typically offer NETD values between 30 and 130 mK, whereas imagers with cooled detection units have an NETD value below 20 mK.

During the acquisition process of thermal infrared images, two cameras were used with parameters described in Table 1. The first camera was a state-of-the-art stand-alone imager, while the second one was a low-cost mobile camera attached to a smartphone. The selected imagers provided different performances thus the results analysis should reveal constraints of using thermal cameras for PAD.

In the paper, faces covered with a printed photograph, displayed photograph, 3D-printed facial mask and full-face latex masks were considered. Spoofing instruments including facial masks, paper sheets or electronic displays were considered as a layer of porous material, partly transmitting thermal radiation through an optical filter or fully blocking thermal radiation. As a result of the presentation attack less thermal energy radiated by the face reached the thermal infrared imager during the measurement.

Theoretically, according to Planck’s law, each body being in the thermal equilibrium emits the radiation in a broad spectral range [26]. The heat conduction is described by Fourier’s law, which states that the time rate of a heat transfer through a material is proportional to the negative gradient in the temperature and to the area, at right angles to that gradient, through which the heat flows *q* = −*k∇T*, where *q* is the local heat flux density, k is the material’s conductivity (related to temperature) and ∇T is the temperature gradient. Fourier’s law of thermal conduction is often simplified and presented in a one dimensional form of *q* = −*k dT*/*dx*.

Since differences between image pixels correspond to differences of the apparent temperature of objects, detection of the presentation attack relies on an analysis of differences between image pixels or groups of pixels. The presence of PAI introduces a change of heat emission because the heat emitted by the subject’s head is partially or fully blocked and absorbed. The analysis of differences between pixels and groups of pixels in the head region should reveal emission differences caused by the presence of a PAI. Since objects of similar or the same heat emission may not be distinguishable in a thermal image, thermalization of PAIs may influence the PAD performance in the thermal infrared domain. During this study, the thermalization impact on the presentation attack detection performance was investigated.

Since thermalization concerns PAIs having direct contact with a human body, 3D printed masks and full-face latex masks were investigated during long-lasting experiments. During a direct contact, the energy (heat) is transferred between the face and the mask by heat transmission between those two bodies, and those two bodies directly connected strive to obtain thermodynamic equilibrium. Ideally, the temperature of both of them within a certain time equalizes. In the case where one body has a constant temperature (human body) and the other lower ambient temperature, both bodies will tend to balance. As a result, the second body (mask) is heated by taking energy from the first body. The proposed measurement methodology aims to analyze the impact of heating of masks on PAD performance.

### 3.2. Dataset Collection

During this study, a TFSD database of attacks using a printed photograph, displayed photograph, 3D-printed facial and full-face latex masks was recorded.

**Figure 1:**
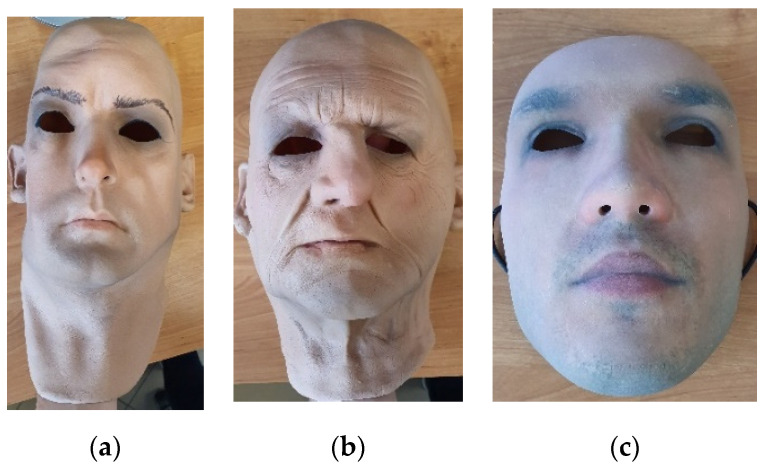
Images of latex masks (**a**,**b**) and a 3D-printed mask (**c**).

Full-face flexible masks are made of foam-latex and present realistic human faces. The masks cover the entire head together with the neck. Sample images of latex masks are shown in Figure 1a,b. The inner surface of masks is an adhesive, thus the masks may hold well when worn. Masks are customized, they can be manufactured in various sizes, thus could be dedicated to each subject. The masks are manufactured with holes in eye, nose and mouth locations. During the study 7 different masks of various sizes and different appearance were used.

The second type of masks used during the study was manufactured by Thatsmyface. The 3D-printed masks are inflexible, thus they do not fully adhere to facial shapes. The masks were customized and printed based on a 3D model generated from 2D photographs of the subject’s face. Each mask covers the front of the face and is attached with a tape. The size of the masks was standardized. During the study three different masks presenting four subjects were used. Sample image of a 3D-printed mask is presented in Figure 1c.

In order to assess the thermalization impact, 10 subjects wearing 3D-printed facial masks and full-face latex masks were asked to wear the mask for 10 min. For each of the PAIs, two sessions were recorded—at the start of experiment and after 10 min. During experiments, subjects were asked not to take off the mask. All the images were collected indoors with subjects presenting faces in front of the camera at a distance of around 1.5 m. During the experiment a subject was sitting in front of the camera performing specific actions guided by a person supervising the measurement process. Each set of images present the subjects’ face in frontal as well as various head positions (looking down, looking left, looking right and looking up, five images for each head position). For each of the head positions, five images were acquired from two cameras at the same time, including three images collected with high-end and two with low-end camera. In order to assure that the angle of head rotation (around 15 degrees) is always the same, numbered markers were installed on the walls and on the floor of the room. A subject was asked to move or rotate the head towards and look at the marker. As a result, 50 images of each PAI were acquired for each subject (including 25 images at the beginning of experiment and 25 images at the end of experiment).

**Figure 2:**
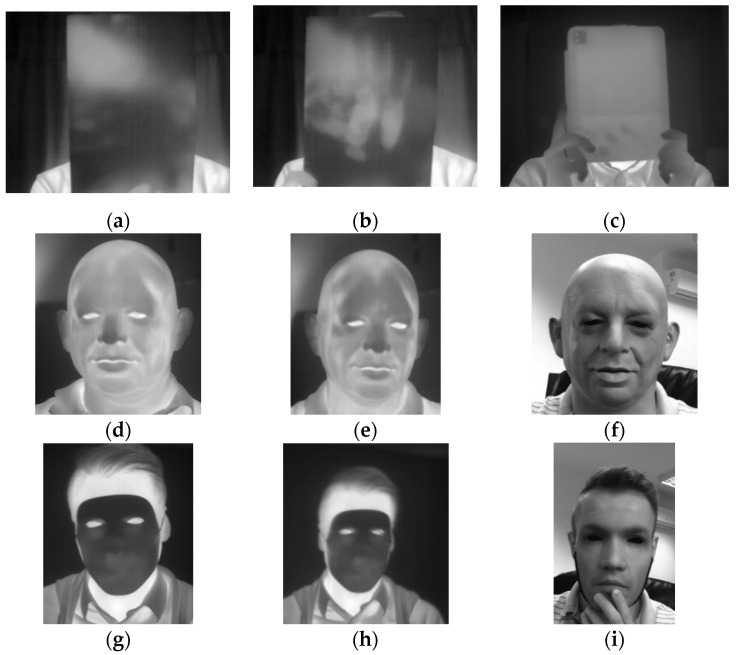
Thermal images presenting a subject face under various presentation attacks, (**a**) face photograph printed on a thin sheet of paper, (**b**) face photograph printed on a sheet of paper with a palm print, (**c**) photograph displayed on an Apple iPad screen, (**d**) subject wearing a latex full-face mask at the beginning of the experiment, (**e**) subject wearing a latex full-face mask at the end of the experiment; (**f**) subject wearing a latex full-face mask, monochrome visible range image, (**g**) subject wearing a 3D printed face mask at the beginning of the experiment, (**h**) subject wearing a 3D printed face mask at the end of the experiment and (**i**) subject wearing a 3D printed face mask, monochrome visible range image.

The A65 camera was operating in the autocalibration mode recording 14-bit depth images while the Compact Pro imager was operated manually recording 8-bit images. A gallery of the presentation attacks is shown in Figure 2.

All the analyses presented in this paper used normalized pixel intensities instead of absolute temperatures since absolute temperatures depend on variable ambient conditions and objects’ emissivity. The normalization method based on a reference maxi-min model is described by Equation (1). Each pixel of the normalized image was calculated based on a reference image. As a reference, a blackbody image with a temperature set up to 295 K was used.

(1) $$x_{k}=x_{k}-\frac{x_{max}-x_{k}}{x_{max}-x_{min}}\cdot \left|\Delta x\right|,$$

where Δx is the difference between image pixel intensity and intensity of the corresponding pixel in a reference image, x_k_ is the pixel’s normalized value and x_max_ and x_min_ are the maximum and minimum values of intensity in the image, respectively.

**Figure 3:**
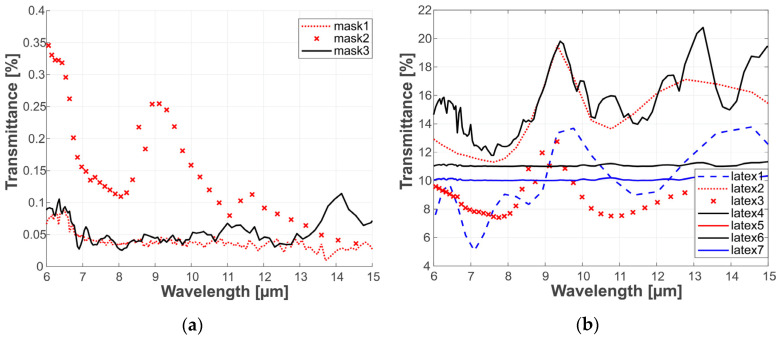
Thermal transmission characteristics of (**a**) 3D- printed masks and (**b**) full-face latex masks in the range of 6–15 mm.

Since the study’s aim regards PAD in a thermal infrared domain, facial masks were characterized in a broad range of infrared radiation to estimate materials’ transmissive capabilities. Characterization was performed using a Fourier transform infrared spectrometer operating in a transmission mode. Transmission graphs are presented in Figure 3.

Infrared transmission significant difference is shown between characteristics of 3D-printed and latex masks. The mean values for latex masks were more than 10 times greater than the mean values for 3D-printed masks. The latex masks were of various thickness, which may influence the transmission uniformity, thus for comparison, all the measurements were taken at the same fragment of the mask. The 3D-printed masks had very low transmittance, which could be considered negligible.

In addition to the in-house dataset, two publicly available datasets, namely CSMAD and MLFP datasets were used during algorithm development and validation.

## 4. Intensity Analysis

The collected images of subjects wearing facial masks were analyzed in order to show changes of pixel intensity during long-lasting measurement sessions. For the purpose of analysis, nine different landmarks were selected as presented in Table 2. Those landmarks were numbered from 1 to 9, where {1,2} are located at the ears, {3,4} describe eye regions, {5} is a nose tip, {6} is a mouth (lips) and {7,8,9} are three landmarks located at the neck region. Since the two types of masks covered different parts of the head, these landmarks were selected to address bare facial points, as well as points at a mask.

**Figure 4:**
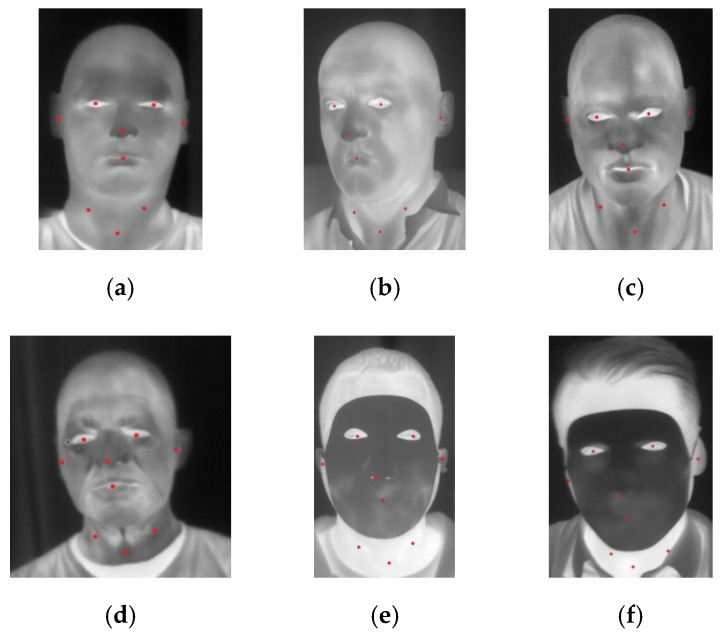
Annotated thermal images presenting the subject wearing (**a**,**b**) flexible latex masks acquired using a high-end camera, (**c**,**d**) flexible latex masks acquired using a low-cost camera and (**e**,**f**) 3D-printed mask acquired using a high-end camera. All images annotated, indicating analyzed landmarks.

**Figure 5:**
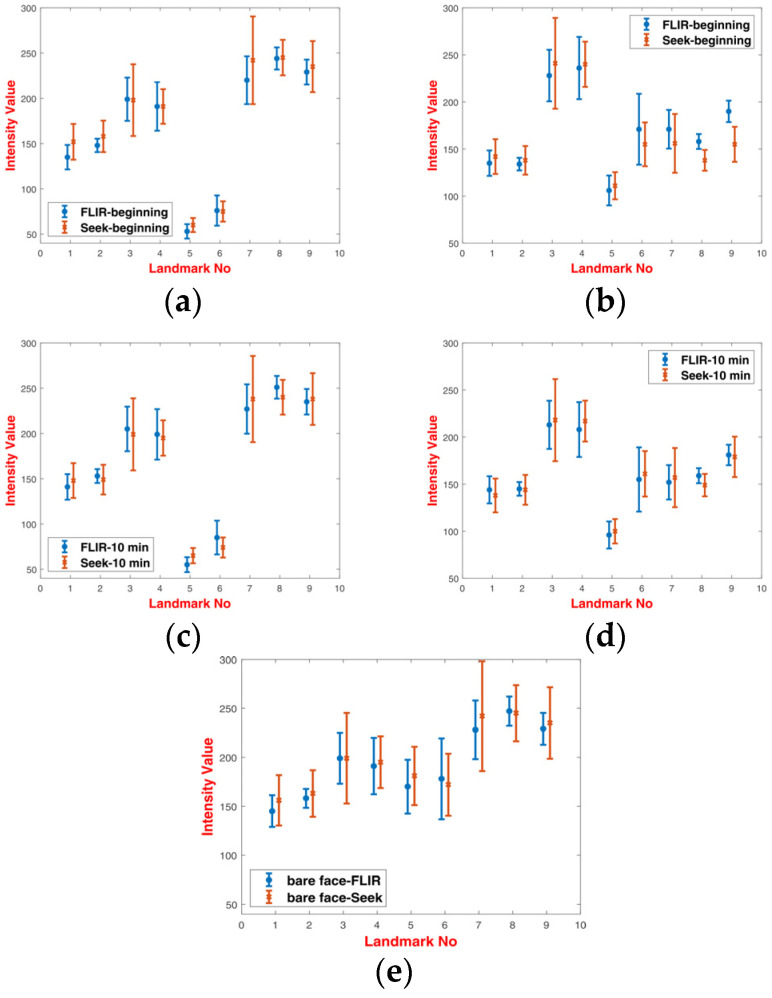
Graphs of the mean values of intensities with minimum and maximum values for two experiment sessions, (**a**) subjects wearing a 3D-printed mask at the beginning of the experiment, (**b**) subjects wearing a latex mask at the beginning of the experiment, (**c**) subjects wearing a 3D-printed mask after 10 min of the experiment, (**d**) subjects wearing a latex mask after 10 min of the experiment and (**e**) a bare face.

For the purpose of analysis, 200 images equally distributed between 3D-printed and latex masks were selected and manually annotated. Sample annotated images are presented in Figure 4. Graphs of normalized intensities for chosen landmarks are presented in Figure 5.

Analysis of intensities for respective landmarks showed that latex masks heated up very quickly during the experiment, and there was a small difference between the values at the beginning and at the end of experiments (after 10 min). On the other hand, 3D printed masks heated up much slower. It was revealed that there was a noticeable difference of intensities between landmarks covered with a 3D printed mask and those uncovered, especially for landmarks {5,6} and {7,8,9}. Latex masks, due to higher transmittance distributed thermal energy in a more uniform way.

When comparing these two types of masks, it was noticed that masks of lower transmission were more distinct from a bare human face. As a result, masks with high transmission, i.e., latex masks, were more difficult to detect than the low-transmittance masks.

## 5. PAD Method

The collected data, as well as images taken from other publicly available datasets were used to develop the presentation attack detection method. During the study, 16 different algorithms were investigated including eight algorithms for head detection and eight algorithms for image classification.

The in-house dataset was enriched with CSMAD and MLFP datasets. A short characterization of both datasets is presented below.

(1) The MLFP dataset consists of images collected from 10 subjects wearing 7 different latex masks and 2D paper masks recorded in visible, near-infrared and thermal infrared spectrum. While six masks cover the entire face, the seventh mask is a half-mask that covers the face region below the eyes. 2D paper masks are created using high resolution images on a high quality card paper with cutouts for eyes. The database contains 1350 videos in total, out of which 1200 videos are attack videos and 150 videos are real access videos. The database consists of 10 subjects (4 females and 6 males) between the ages of 23–38 years. The videos have been captured in indoor and outdoor conditions. Each image extracted from the videos has a pixel resolution of 640 × 480. Thermal resolution of the imager and thermal transmission of PAIs are not provided.
(2) The CSMAD dataset contains images of presentation attacks made of six custom-made silicone masks collected from six different subjects. The dataset contains images with a resolution of 320 pixels × 240 pixels acquired using Seek Thermal Compact Pro. Attacks have been recorded for all six targets, made by different subjects under different illumination conditions.

**Figure 6:**
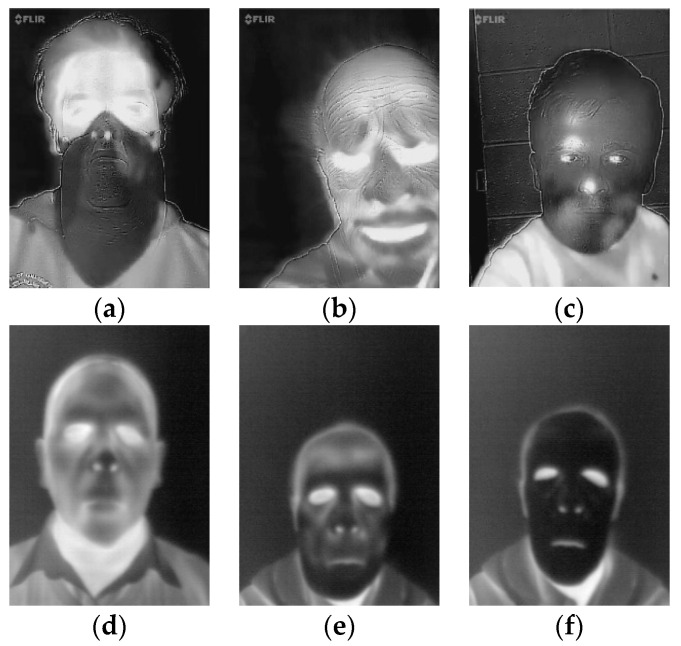
Gallery of images from (**a**,**b**) the MLFP database, latex masks (**c**) MLFP database, 2D paper mask and (**d**–**f**) CSMAD database.

Incorporation of more datasets allowed one to enlarge the set of PAIs. Overall, the range of PAIs considered during development of the PAD method consists of printed 2D photographs, displayed 2D photographs, 2D paper masks, 3D-printed masks, silicone masks and 3D full-face latex masks. A gallery of sample images from MLFP and CSMAD datasets is presented in Figure 6. The entire dataset used during algorithm development consisted of 3500 images presenting genuine subjects and 3500 images presenting attacks with various PAIs. The total number of images per type of mask has been provided in Table 3.

For the purpose of algorithm development, two datasets [25,27] of thermal images presenting genuine subjects were used. In order to validate the method against changing subject’s state, images of subjects after physical effort were added.

**Figure 7:**
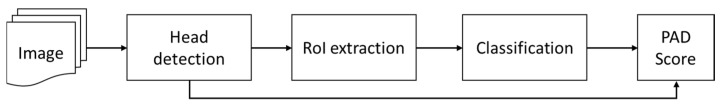
Block diagram of the presentation attack detection method.

The proposed method detects attacks in a two-step scenario. The first step of the algorithm aims to detect the head and to determine coordinates of a region of interest (RoI) corresponding to the head. In the second step, a trained classification algorithm performs a classification of the detected RoI. The general scheme of the PAD method is presented in Figure 7.

### 5.1. Head Detection Algorithm

Object detection algorithms trained to detect a human head in a thermal infrared domain has been incorporated as the first step of the proposed method. The head detection algorithm plays two roles depending on the PAI type. First, the head detector determines whether a human head is presented to the camera. It applies to all the situations and the subject’s head is visible in the image. However, in the case of attacks where the face is fully shielded, thus not visible in the image, the head detector may play a role of the presentation attack detector.

When presenting a facial photograph, printed or displayed in front of a thermal camera, the energy radiated by the subject’s face is blocked by the PAI due to a negligible transmission of thermal energy through paper. As a result an acquired image contains a thermally uniform rectangle instead of a facial thermogram as presented in Figure 2a–c. The thermal image of a printed photograph or a tablet may contain thermal spots, which correspond to thermal remaining of fingers or palm prints. In the case of facial masks, the head detector provides coordinates and size of the head region bounding box.

Several algorithms were investigated for the head detection task. The selected methods present two different approaches to object detection—YOLO v3 [28] as a single-shot detector (SSD) and Faster R-CNN [29] as a region-proposal network (RPN).

The Faster R-CNN algorithm is based on the idea of RPN. RPN outputs the objectness score for many proposed boxes, which indicates whether the image selected part contains a background or a foreground object. All the boxes were examined by a classifier and a regressor to check the objects’ occurrence. Faster R-CNN is composed of a feature extraction network and two subnetworks: region proposal network for generating region proposals and a network using these proposals to detect objects. Four state-of-the-art pretrained deep neural networks were used for feature extraction. For comparison purposes, the same set of networks was selected for YOLO and Faster R-CNN, including ResNet-50 [30], ResNet-101 [30], GoogLeNet [31] and Inception v3 [32].

YOLO is known as a very fast object detection algorithm. For this study, the third iteration of the algorithms was investigated. The YOLO v3 object detection network was composed of two subnetworks—a feature extraction network followed by a detection network. Four feature extraction networks were used including ResNet-50 [30], ResNet-101 [30], GoogLeNet [31] and Inception v3 [32]. The object detection process relies on a single CNN that simultaneously predicts multiple bounding boxes and class probabilities for those boxes. YOLO considers object detection as a single regression problem, straight from image pixels to bounding box coordinates and class probabilities. The network uses batch-normalization to normalize the outputs of the hidden layers and anchor-boxes to predefine the size of bounding box, thus improving detection performance. The classification is done with independent logistic classifiers in order to calculate the likeliness of the input belonging to a specific label. It predicts all bounding boxes across all classes for an image simultaneously. During image processing, the image is taken globally to make predictions.

All the head detection algorithms were first trained on the ImageNet dataset and retrained with thermal infrared and visible range face images presenting both genuine subjects, as well as spoofed samples.

### 5.2. Deep Learning Classifiers

The head images extracted from original images were passed to a deep neural network for presentation attack detection. Eight state-of-the-art CNNs were investigated during this study including residual networks in various options, inception networks in different variants and a network dedicated for mobile devices. A set of selected deep learning models consists of ResNet-18 [30], ResNet-50 [30], ResNet-101 [30], GoogLeNet [31], Inception v3 [32], MobileNet-2 [33], DenseNet-201 [34] and Xception [35].

Each deep neural network was trained to analyze heat maps of thermal images and to classify as genuine or an imposter. All the selected networks were first trained on ImageNet dataset and retrained on a variety of images presenting genuine samples, as well as spoofed samples. For training purposes, the genuine and spoofed samples including front-facial images, as well as images presenting tilted head were used.

## 6. Validation

During validation two PAD metrics were used, namely the attack presentation classification error rate (APCER) and bona fide presentation classification error rate (BPCER). APCER corresponds to the proportion of attack presentations using the same PAI species incorrectly classified as bona fide presentations in a specific scenario while BPCER corresponds to the proportion of bona fide presentations incorrectly classified as attack presentations in a specific scenario. During training, several parameters have been set up as provided in Table 4.

### 6.1. Head Detection Algorithm

First, performance of the head detection algorithm was validated against attacks where the human head was not visible in an image. This concerns a detection of 2D printed or displayed photographs. Results of head detection with regard to printed and displayed photographs are presented in Table 5.

The main finding was that both printed and displayed attacks were detectable with similar error rates. Error rates of all four models based on YOLO architecture were larger than 1.5% whereas models based on Faster R-CNN architecture offered a zero error rate across validation datasets.

As the second step, the head detector was validated against attacks using all the remaining PAIs—2D paper masks, 3D-printed, silicone masks and 3D full-face latex masks. The dataset was divided into training, test and validation sets with a split ratio of 70% (4900 images), 10% (700 images) and 20% (1400 images), respectively. All the algorithms were evaluated with regard to the detection rate (number of correct detections per testing dataset) and false detection rate (number of images for which false detections occurred per testing dataset). Results are presented in Table 6.

Both object detection methods achieved high head detection rates and low false detection rates. Results were almost equal across all PAIs, however the lowest scores were achieved for 2D paper mask attacks. Generally, the Faster R-CNN algorithm achieved better results than YOLO in all configurations. The best performing version of YOLO used Inception v3 for feature extraction and obtained results very close to the results obtained by Faster R-CNN in the same configuration.

Both object detection methods achieved high head detection rates and low false detection rates. Results were almost equal across all PAIs, however the lowest scores were obtained for 2D paper mask attacks. Generally, Faster R-CNN algorithm obtained in all configurations better results than YOLO. The best performing YOLO version used Inception v3 for feature extraction and was very close to results obtained by Faster R-CNN in the same configuration.

### 6.2. Deep-Learning Classifiers

Two training and validation approaches were applied for deep learning classifiers. In the first approach, a ten-fold cross-validation method was applied. A random selection of 70% of all images was used as the training set (4900 images) while the remaining 30% constituted the testing and validation sets with a split ratio of 10% (700 images) and 20% (1400 images), respectively. The mean results of the 10-fold cross-validation are presented in Table 7.

**Figure 8:**
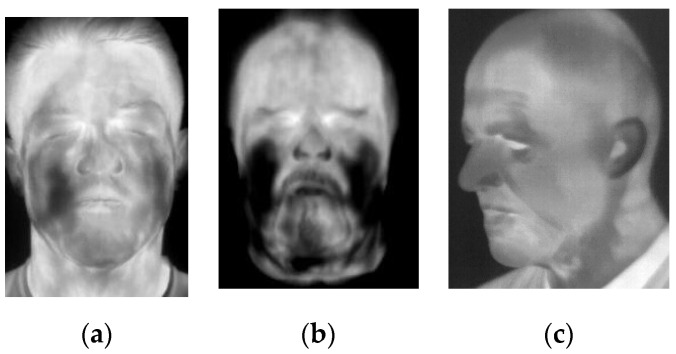
Sample images used during validation: (**a**,**b**) genuine subjects classified as a presentation attack and (**c**) presentation attack classified as a genuine subject.

The ten-fold cross validation revealed an impressive performance of all eight investigated neural networks. The investigated methods achieved a high performance when trained on all known materials. Six of the eight classifiers achieved zero error rates. The remaining two neural networks (NN), GoogLeNet and ResNet-18 achieved error rates below 0.1. The results’ analysis revealed that these two NNs have failed to correctly detect attacks when the subject’s head is tilted as presented in in Figure 8c. Moreover, some genuine samples have been classified as attacks. These samples present subjects after physical effort and are presented in Figure 8a,b. The subject’s face changed significantly after physical effort. When the face started to sweat, the apparent temperature registered by a thermal imager decreased and the face thermal image was not uniform.

As the second validation approach, the cross-material validation was applied to verify the proposed method generalization. During the cross-material validation, training and testing split were based on known and unknown materials. In all the cross-material scenarios, graphs of reflectance in the frequency domain of one group of materials were partitioned to the classifier for training, and the spoof impressions of the second group of “unknown” materials were kept aside for testing. The images collected during long-lasting experiments with 3D-printed and full-face latex masks were validated separately to assess a thermalization impact on PAD performance. In this manner the generalization capability of the spoofing detection method was assessed. Results of cross-material validation are presented in Table 8 and Table 9.

The cross-material experiments on the deep learning models revealed that PAD performance was not uniform across all materials. Latex and silicone masks generalized very well to 3D printed and 2D paper masks. Thermalization impact on PAD performance for attacks using 3D-printed masks was low, the scores at the beginning and end of experiment sessions were similar. The highest error rates were noticed for models validated on full-face latex masks taken from TFSD. Moreover, validation on images collected at the beginning and end of experiment revealed that the performance decreased for attacks using masks heated during the experiment. These results were of great importance since in real-life scenarios masks were worn long before approaching the PAD system. It seems that full-face latex masks were the most difficult to detect as they were characterized with a high heat transmission and covered the entire head. Thermal energy transfer through full-face latex masks was more uniform than through the 3D-printed masks, which made the detection more difficult.

### 6.3. Discussion

The common feature of all the attacks was that each presentation attack instrument introduced a change of heat emission of the facial or head region. The presented method takes advantage of the heat emission disturbance introduced by PAIs. From the results in previous subsections, it could be seen that both 2D and 3D attacks could be efficiently detected.

In the k-fold validation scenario, the investigated algorithms provided impressive performance. Detection of 2D presentation attacks can be performed with a zero error rate to APCER of 3.9% depending on an object detection framework and a feature extraction network. A ten-fold cross-validation of classifiers shows zero error rates for almost all neural networks. Experiments in the unseen attack scenario show some disproportions between the detection performance of deep learning models across different attacks with the full-face latex masks being the most difficult to detect.

In contrary to other works focused on algorithms, the algorithms used for the purpose of this study were state-of-the-art image classifiers or object detectors, not tailored for specific attacks. The presented method surpassed the selected feature-based and CNN-based baselines. The block-based LBP features with LDA [18] for detection of 2D attacks and 3D printed masks using visible and 3D imagery achieved HTER values of 0.95% and 1.27%, respectively. PAD methods [21] based on FaceNet, LightCNN and VGG-Face using visible light imaging achieve impostor’s attack presentation matches rates of 22.6%, 42.1% and 57.9%, respectively. For comparison, the approach based on thermal infrared and visible spectra [23] with the PCCW-CCA method achieved a detection rate of 85.1% and 90.8% for data including and excluding glasses with a false acceptance rate of 0.1%. The Thermal Face-CNN [24] obtained the best accuracy of 79.18%, which was far below the proposed method.

The best performing methods connect various imaging modalities including the visible range, depth, infrared and thermal infrared images. The multi-channel convolutional neural network (MC-CNN) [13] achieved impressive results in a k-fold validation as well as in an unknown attack scenario. This method was reported to obtain an ACER of 2.59% for thermal imaging and 0.84% for a combination of grayscale, depth, infrared and thermal infrared, respectively.

The long-lasting experiments as well as experiments with various subject’s physical states aimed to show real-life situations. From results shown in previous subsections it can be seen that performance of PAD algorithms generally decreased as a result of the thermalization and physical effort of a subject.

It has to be underlined that other works do not take into account the impact of variable subject’s state and heat transfer between subject’s face and the mask on the PAD performance. Therefore, the real-life results may differ from those reported in papers mentioned above.

It should be highlighted that high-end and low-cost cameras used in this study offered relevant parameters for the attack detection. Both head detectors and deep-learning classifiers achieved similar results independently from a camera providing an image for the attack detection. This remark leads to the conclusion that the PAD in the thermal infrared domain could be efficiently performed using low-cost, affordable sensors.

## 7. Conclusions

This study concerned the detection of various presentation attacks in thermal infrared. The process of detecting facial spoofing attempts may have a different physical sense in various light spectra and may be influenced by various factors. The ability to measure and visualize head heat distribution in a thermal infrared domain seems very solid for achieving robust presentation attack detection capability.

This paper considers real-life attacks, where the subject’s state may be changed by physical effort. The study also presents the impact of thermalization on PAD performance. Long lasting experiments with printed and displayed photographs, 3D-printed and full-face latex masks were performed. Images have been analyzed to reveal the impact of masks’ thermalization. The long lasting experiments revealed that the thermalization influenced the performance of presentation attack detection. The performance of PAD algorithms generally decreased for attacks using masks heated during the experiment. This is of great impact for real life scenarios, when a subject wears PAI for some time before being screened. It has been also noticed that the subject’s physical state is influencing the PAD performance. Genuine subjects after physical effort were frequently classified as attack presentations. This is very important, as thermal face emission changes as a result of stress, physical effort or illness.

The spoofing masks were spectrally characterized. Collected data including attacks composed of the thermal face spoofing dataset (TFSD) that is published along with this manuscript. The collected dataset was enriched with two publicly available datasets. Finally, during this study presentation attacks with the use of printed photographs, displayed photographs, 2D paper masks, 3D-printed masks and 3D full-face latex masks were investigated.

A two-step presentation attack detection method was proposed. The method was composed of a head detection algorithm and deep neural network classification algorithm. The presented method allowed us to detect all types of attacks considered in this study, including attacks with simple 2D photographs and customized masks.

Validation results of 16 state-of-the-art algorithms were presented. Classification algorithms provided an impressive performance in a k-fold cross-validation scenario. However, the cross-material validation revealed disproportions between detection performance of deep learning models across different attacks. It seems that full-face latex masks were the most difficult to detect as they transmit heat efficiently and uniformly across the entire head.

Results of this study together with the dataset will be used for further investigations on new PAD algorithms. Further works will be directed to develop algorithms more suitable for thermal intensity imaging. Moreover, investigations on use of possible new PAIs are foreseen.

## Figures and Tables

**Table 1:** Imager parameters.

Parameters	Parameters	
	FLIR A65	Seek Thermal Compact Pro
Detection unit	FPA (focal plane array), microbolometer array	FPA (focal plane array), microbolometer array
Resolution	640 pixels × 512 pixels	320 pixels × 240 pixels
spectral range	7.7 μm–11.5 μm	7.5 μm–12 μm
NETD	<50 mK	<80 mK

**Table 2:** Landmarks numbering.

Landmark No	Landmark Location
1, 2	ears
3, 4	eyes
5	nose tip
6	mouth
7, 8, 9	neck

**Table 3:** Number of images in respective datasets.

Type of PAI	Number of Images
**3D-printed mask**	250 ^1^/250 ^2^
**Latex mask**	500 ^3^250 ^1^/250 ^2^
**Silicone mask**	500 ^4^
**Printed photograph**	500 ^5^
**Displayed photograph**	500 ^5^
**2D paper mask**	500 ^3^
**Genuine**	2500/1000 ^6^

^1^ Images from in-house database, beginning of measurement session. ^2^ Images from in-house database, end of measurement session. ^3^ Images from MLFP. ^4^ Images from CSMAD database. ^5^ Images from in-house database. ^6^ Images of genuine subjects after physical effort.

**Table 4:** Training parameters of head detectors.

Parameter	Methods		
	YOLO	Faster R-CNN	Classifiers
Learning rate	10^−3^	10^−4^	3 × 10^−3^
Maximum no. of epochs	100	100	100
Batch Size	10	1	10
Optimizer	stochastic gradient descent with momentum		

**Table 5:** Validation results of the head detector as a presentation attack detector ^1^.

Method	Printed Photograph		Displayed Photograph	
	APCER	BPCER	APCER	BPCER
YOLO v3 + ResNet 50	3.51	3.44	3.51	3.44
YOLO v3 + ResNet 101	3.90	3.73	3.22	3.09
YOLO v3 + GoogLeNet	3.90	3.73	1.89	1.76
YOLO v3 + Inception v3	1.73	1.66	0.00	0.00
Faster R-CNN + ResNet 50	0.00	0.00	0.00	0.00
Faster R-CNN + ResNet 101	0.00	0.00	0.00	0.00
Faster R-CNN + GoogLeNet	0.00	0.00	0.00	0.00
Faster R-CNN + Inception v3	0.00	0.00	0.00	0.00

^1^ All the values are given in %.

**Table 6:** Validation results of head detector ^1^.

Method	3D-Printed		Latex		Silicone		2D Paper	
	Det. Rate	False Det. Rate	Det. Rate	False Det. Rate	Det. Rate	False Det. Rate	Det. Rate	False Det. Rate
YOLO v3 + ResNet 50	0.97	0.01	0.97	0.01	0.98	0.01	0.94	0.01
YOLO v3 + ResNet 101	0.99	0.01	0.99	0.01	0.99	0.01	0.93	0.01
YOLO v3 + GoogLeNet	0.99	0.00	0.99	0.00	0.99	0.00	0.98	0.00
YOLO v3 + Inception v3	1.00	0.00	1.00	0.00	1.00	0.00	0.99	0.00
Faster R-CNN + ResNet 50	0.99	0.01	0.99	0.01	0.98	0.01	0.96	0.01
Faster R-CNN + ResNet 101	1.00	0.01	1.00	0.01	1.00	0.01	0.97	0.01
Faster R-CNN + GoogLeNet	1.00	0.00	1.00	0.00	1.00	0.00	0.98	0.00
Faster R-CNN + Inception v3	1.00	0.00	1.00	0.00	1.00	0.00	1.00	0.00

^1^ Absolute values are given in the range between 0 and 1.

**Table 7:** Results of 10-fold cross-validation.

Method	All	
	APCER ^1^	BPCER ^1^
GoogLeNet	0.03 ± 0.01	0.03 ± 0.01
Inception v3	0.00	0.00
ResNet-18	0.06 ± 0.01	0.04 ± 0.01
ResNet-50	0.00	0.00
ResNet-101	0.00	0.00
MobileNet-2	0.00	0.00
DenseNet-201	0.00	0.00
Xception	0.00	0.00

^1^ All the values are given in %.

**Table 8:** Results of cross-material validation ^1^.

Method	3D-Printed Mask ^2^		3D-Printed Mask ^3^		TFSD Latex Mask ^4^		TFSD Latex Mask ^5^	
	APCER	BPCER	APCER	BPCER	APCER	BPCER	APCER	BPCER
GoogLeNet	0.00	0.00	0.00	0.00	11.11	10.62	13.09	12.60
Inception v3	1.51	1.45	1.51	1.45	31.02	30.38	34.02	33.22
ResNet-18	3.82	3.55	3.90	3.73	37.04	37.04	40.14	39.11
ResNet-50	1.43	1.40	1.73	1.66	21.49	21.49	24.41	23.79
ResNet-101	3.38	3.23	3.68	3.52	23.11	23.11	26.08	26.51
MobileNet-2	1.28	1.11	1.08	1.03	6.05	6.05	9.15	9.01
DenseNet-201	1.43	1.34	1.73	1.65	7.66	7.26	10.34	10.01
Xception	1.11	1.05	1.08	1.03	6.05	6.05	9.12	8.90

^1^ All the values are given in %. ^2^ Images collected at the beginning of the experiment. Models trained on all masks except 3D-printed. ^3^ Images collected at the end of the experiment. Models trained on all masks except 3D-printed. ^4^ Images collected at the beginning of the experiment. Models trained on all masks except latex masks. ^5^ Images collected at the end of the experiment. Models trained on all masks except latex masks.

**Table 9:** Results of cross-material validation ^1^.

Method	Silicone Mask		2D Paper Mask		MLFP Latex Mask	
	APCER	BPCER	APCER	BPCER	APCER	BPCER
GoogLeNet	4.50	4.49	1.08	1.03	5.30	5.22
Inception v3	4.50	4.49	1.73	1.66	5.53	5.11
ResNet-18	8.69	8.67	3.90	3.73	6.64	6.43
ResNet-50	30.10	30.98	1.51	1.45	11.11	11.00
ResNet-101	7.19	7.17	3.68	3.52	6.13	6.01
MobileNet-2	8.51	8.48	1.51	1.45	9.55	9.13
DenseNet-201	0.00	0.00	1.73	1.65	0.10	0.10
Xception	4.50	4.49	1.08	1.03	3.50	3.33

^1^ All the values are given in %.
